# Face Masks Protect From Infection but May Impair Social Cognition in Older Adults and People With Dementia

**DOI:** 10.3389/fpsyg.2021.640548

**Published:** 2021-08-13

**Authors:** Matthias L. Schroeter, Jana Kynast, Arno Villringer, Simon Baron-Cohen

**Affiliations:** ^1^Department of Neurology, Max Planck Institute for Human Cognitive and Brain Sciences, University Hospital Leipzig, Clinic for Cognitive Neurology, Leipzig, Germany; ^2^Department of Psychiatry, Autism Research Centre, University of Cambridge, Cambridge, United Kingdom

**Keywords:** coronavirus, COVID, dementia, emotion recognition, face masking, mindreading, social cognition

## Abstract

The coronavirus disease 2019 (COVID-19) pandemic will have a high impact on older adults and people with Alzheimer's disease and other dementias. Social cognition enables the understanding of another individual's feelings, intentions, desires and mental states, which is particularly important during the COVID-19 pandemic. To prevent further spread of the disease face masks have been recommended. Although justified for prevention of this potentially devastating disease, they partly cover the face and hamper emotion recognition and probably mindreading. As social cognition is already affected by aging and dementia, strategies must be developed to cope with these profound changes of communication. Face masking even could accelerate cognitive decline in the long run. Further studies are of uppermost importance to address face masks' impact on social cognition in aging and dementia, for instance by longitudinally investigating decline before and in the pandemic, and to design compensatory strategies. These issues are also relevant for face masking in general, such as in medical surroundings—beyond the COVID-19 pandemic.

## Introduction

**Figure 1:**
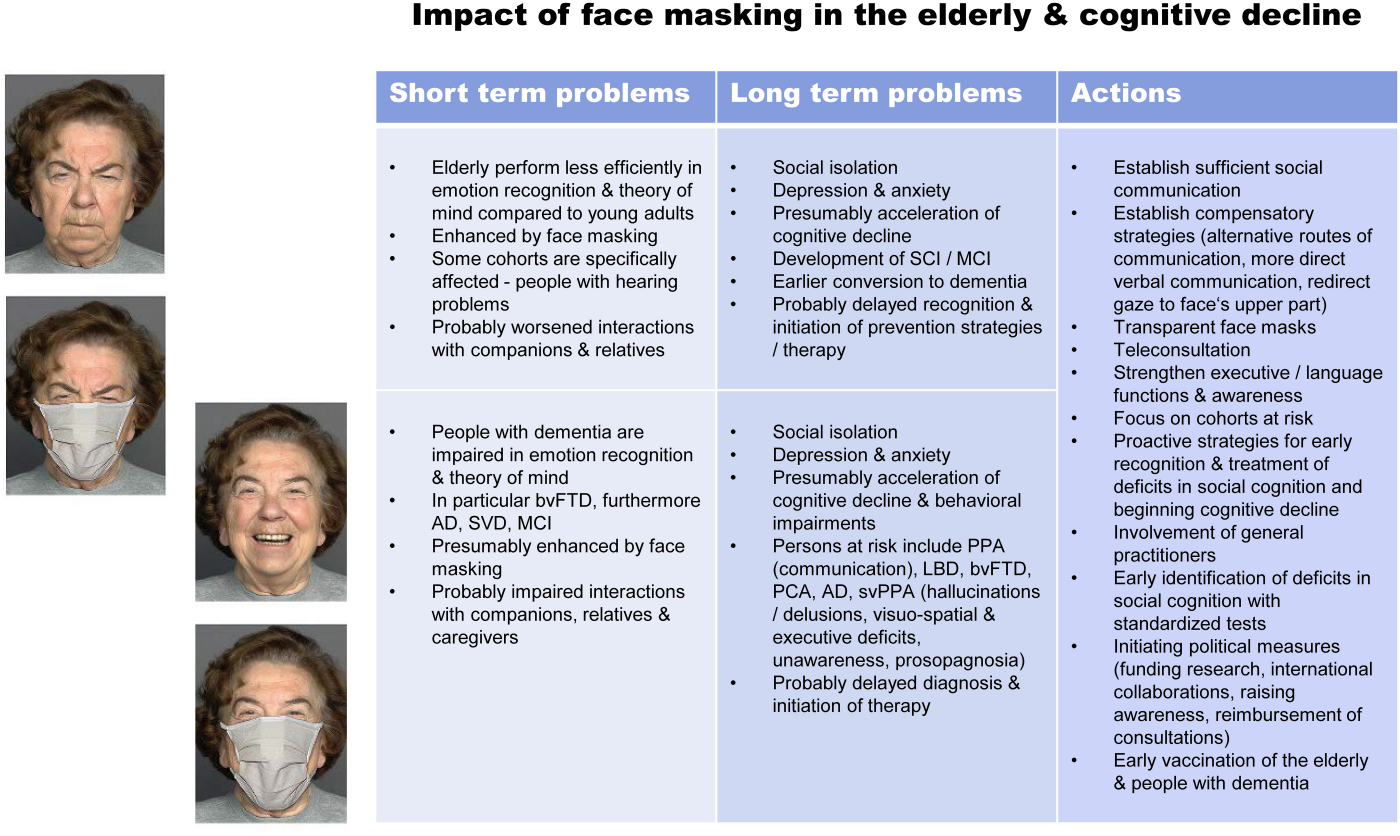
Impact of face masking on social cognition in older adults (top) and people with dementia (bottom) and possible actions to encounter these problems. Photographs represent stimuli (angry and happy) from Carbon (2020). Further respective images can be found in the FACES database (Ebner et al., 2010; https://faces.mpdl.mpg.de/imeji/). Carbon (2020) and Ebner et al. (2010) gave permission for publication of these images in the article. AD, Alzheimer's dementia; bvFTD, behavioral variant frontotemporal dementia; LBD, Lewy body dementia; MCI, mild cognitive impairment; PCA, posterior cortical atrophy; PPA, primary progressive aphasia; SCI, subjective cognitive impairment; SVD, small vessel disease; svPPA semantic variant primary progressive aphasia.

Social cognition requires, beside others, the understanding of another individual's feelings, intentions, desires and mental states, coined theory of mind or mindreading (Baron-Cohen et al., 2001). Successful social cognition is even more important during extraordinary situations such as the coronavirus disease 2019 (COVID-19) pandemic that has becoming a devastating sanitary and global emergency (Chen et al., 2020). In particular, handling such a situation necessitates optimal communication to understand the situation and related measures, and close cooperation between people. To prevent further spread of the disease several countries and authorities such as the World Health Organization (WHO) and the US Centers for Disease Control and Prevention (CDC) recommended using face masks among health-care workers and the general public (Greenhalgh et al., 2020; Peeples, 2020). This action intends protecting people from infection with the severe acute respiratory syndrome coronavirus type 2 (SARS-CoV-2), from severe illness, and aims at reducing the need for isolation and quarantining. Here we want to discuss the impact of face masking on social cognition—with a focus on emotion recognition and theory of mind—in older adults and people with dementia with an emphasis on short- and long-term effects, and suggest actions to encounter these problems (for an overview and summary see Figure 1).

## Impact of Face Masking on Social Cognition

Although justified for disease prevention face masks cover parts of the face. As discussed by Carbon (2020) face masks or community masks shielding the mouth and the nose hide about 60–70% of the face area relevant for emotional expression, where the mouth region is of particular importance for emotion recognition. This author investigated the impact of face masking—with typical masks worn during the COVID-19 pandemic—on emotion recognition in a study (for example stimuli see Figure 1).

Face masks severely impaired the performance of assessing the emotional state of a face and the confidence of one's own assessment with medium/large effect size, respectively. Effects appeared emotion-specific in accordance with previous studies showing that the recognition of happiness and sadness, and to a smaller degree anger, rely strongly on the lower face, in particular the mouth. Recognition of disgust was also severely impaired by face masking. Only neutral and fearful faces could be detected in spite of masking, where it is well-known that for fear the eye region provides generally most relevant information for correct recognition. The author further discusses that aging might even boost this masking effect as older adults have more difficulty in recognizing some basic emotions such as disgust, happiness, and fear, and also problems in recognizing other basic emotions such as anger and sadness. However, as a limitation of the study, Carbon's (2020) study relied on visual static stimuli only. Accordingly, these results for static facial emotion recognition have to be confirmed with other stimuli and in other settings. Additionally, the impact of face masking on theory of mind has still to be tested.

## Social Cognition in Aging

Another study by Chaby et al. (2017) investigated gaze strategies accompanying facial emotional processing with aging. During looking at basic facial expressions gaze movements were recorded. Older adults performed worse than younger adults in identifying facial expressions, except for joy and disgust. Remarkably, older adults used a focused-gaze strategy as they focused their attention only on the lower part of the face. Contrary, younger adults used an exploratory-gaze strategy, repeatedly visiting the upper and lower facial areas. This finding is consistent with result of a meta-analysis showing that older adults display a bias to look more at the mouth and less at the eyes in facial emotion processing compared to younger adults (Grainger and Henry, 2020). Hence, older people might lack expertise in exploring upper face regions, which is necessary when the lower face is covered by a mask.

Beside emotion recognition, as shown at least for static facial emotions, other abilities for social cognition might be affected. With face masking people may rely on information from the eyes to attribute mental states to others and successfully interact with them, although in natural settings alternative ways of compensation involve also contextual information, body language, gestures, language, and non-verbal oral information. The “Reading the Mind in the Eyes” Test (Baron-Cohen et al., 2001) seems to be a good proxy to investigate social cognition abilities based on the eye region as it investigates mindreading ability from gaze. This test shows photographs of the eye-region of a person, where the term most appropriately describing the pictured mental state shall be selected. It is focused mainly on “affective” theory of mind, which enables understanding of others' emotions, affective states, or feelings (Henry et al., 2013). Recently, we investigated mindreading ability from gaze with this test in a very large population-based cohort including 1,603 persons aged between 19 and 79 years (Kynast et al., 2020).

The study revealed a linear relation between individual test performance scores and age for women and men. Performance declined with aging in both, women [*F*_(1, 697)_ = 78.8, *p* < 0.001] and men [*F*_(1, 904)_ = 90.6, *p* < 0.001], indicating an age-related decline in mindreading ability from the eyes. Effect size for the decline in performance above vs. below an age of 45 years was large in women (g = 0.75), and medium sized in men (g = 0.55; Hedges' g). A linear regression model confirmed that test performance declined with aging, and showed an association with lower verbal intelligence but not gender.

One might criticize that the “Reading the Mind in the Eyes” Test has limitations. Firstly, its stimulus material shows potential interactions between gender/sex, age, and emotional valence (Kynast and Schroeter, 2018). Generally cognitive tests shall be adapted to the age range of the cohort of interest, here for older adults. Secondly, the “Reading the Mind in the Eyes” Test investigates a very specific and rather artificial situation—mindreading only from the eyes—as in real life other information to infer mental state will be available from context, body language, gestures, language, and non-verbal oral information. Thirdly, Quesque and Rossetti (2020) recently have questioned the specificity of the “Reading the Mind in the Eyes” Test together with several other classic measures for theory of mind. Here, these authors also referred to a debate in the literature regarding the assumed processes assessed with this test, i.e., mental states inference vs. eye-region emotion recognition.

One might have a look at comprehensive meta-analyses to place this test into the framework of social cognition. Indeed, meta-analyses have confirmed effects of age on social cognition for both, “affective” and “cognitive” theory of mind, where the latter enables understanding of cognitive states, beliefs, thoughts, or intentions (see Henry et al., 2013). Note that this comprehensive meta-analysis involved 1,462 participants and several types of theory of mind tasks, i.e., inferring mental states from the eyes, stories where a character's behavior can be best understood by positing an underlying mental state, identification of emotions and cognitive states from video clips, understanding protagonist's false belief, and recognizing social gaffes (Faux pas). Overall, older adults performed more poorly than younger adults in these tests of theory of mind with moderate effect size (r = 0.41; r = 0.43 for inferring mental states from the eyes). Another meta-analysis by Ruffman et al. (2008) with 1,667 subjects has shown a general age-related decline in emotion recognition in faces, voices, bodies/contexts. Hayes et al. (2020) confirmed this finding in another more recent and more comprehensive meta-analysis on facial emotion recognition in 10,526 older and young adult samples.

## Social Cognition in Dementia

Pronounced impairments in social cognition have been reported in frequent neurodegenerative dementia syndromes according to meta-analytical evidence (recently referred to as neurocognitive disorders; American Psychiatric Association, 2013). In particular behavioral variant frontotemporal dementia is characterized by early and severe deficits in social cognition with a strong effect size (d = 1.79; Cohen's d) as shown in a meta-analysis across several theory of mind tasks such as “Reading the Mind in the Eyes” Test, Faux pas task, sarcasm and first and second order false belief tasks (Bora et al., 2015). Here, the “Reading the Mind in the Eyes” test was one of the most impaired tasks (d = 1.38; Bora et al., 2015). Of note, deficits of social cognition are the core cognitive symptom in this disease beside executive dysfunction as shown in the meta-analysis by Dodich et al. (2021) for emotion recognition and theory of mind. Schroeter et al. (2014) aimed at conceptualizing bvFTD neuroscientifically by relating these behavioral deficits to their disease-specific neural correlates. A “Reading the Mind in the Eyes” Test predicted behavioral variant frontotemporal dementia even better than executive function tests (Schroeter et al., 2018) and indicated conversion to this disease early (Pardini et al., 2013).

Social cognition as tested with theory of mind tasks (for specific tests see above) is also impaired in the most frequent neurodegenerative syndrome, i.e., Alzheimer's dementia (d = 1.15), although deficits are modest compared with general cognitive impairment (Bora et al., 2015) affecting the memory domain firstly (Schroeter et al., 2009). Small vessel disease, a frequent cause of vascular dementia, has also been reported to be associated with severe deficits in social cognition as shown for the “Reading the Mind in the Eyes” Test beside altered attention and memory (Kynast et al., 2018; Schroeter, 2021). Even mild cognitive impairment, with an increased probability converting to dementia in the long-term, has been reported to be associated with significant (medium) impairments in theory of mind (d = 0.63) and facial emotion recognition (d = 0.58; Bora and Yener, 2017).

## Consequences of Face Masking in the Short Term

These findings are of particular interest during theCOVID-19 outbreak as widespread face masking is recommended for disease prevention (Greenhalgh et al., 2020; Peeples, 2020). As discussed before older people and persons with dementia are impaired in mindreading from the eyes and emotion recognition. Notably, those cohorts—old people (Chen et al., 2020) and subjects with dementia (Mok et al., 2020)—are also at important risk for COVID-19 itself and associated mortality. Holmes et al. (2020) and Pfefferbaum and North (2020) have discussed that the COVID-19 pandemic generally will lead to social isolation, emotional distress, and increase the risk for psychiatric illnesses, such as anxiety, depression, self-harm and suicide. High-risk populations such as older adults or people with dementia might be particularly affected by isolation and loneliness, supported by the “digital divide.” Recently, Mok et al. (2020) paid particular attention to the profound impact of the COVID-19 pandemic upon older people with Alzheimer's disease and other dementias. They discussed generally challenges encountered and suggested strategies coping with these issues. In the following, we want to discuss the specific impact generated potentially by face masking in aging and dementia. Note that—to date—no studies exist investigating these issues (beside Carbon, 2020), which is a desideratum for the future. As we want to initiate a new perspective/frontier of investigation here we cannot rely on published results sufficiently in the following sections so far.

### How Does Face Masking Affect Social Behavior?

How may alterations in face emotion processing and presumably mindreading due to face masking affect social behaviors during the pandemic? Obviously, face masking impairs (static) facial emotion recognition (Carbon, 2020), which might be particularly relevant in older adults and persons with dementia already compromised in the sociocognitive domain (Ruffman et al., 2008; Henry et al., 2013; Bora et al., 2015; Kynast et al., 2018; Hayes et al., 2020). If face masks are used by older persons, people with dementia and their companions, relatives and caregivers, reading emotions and the mind from faces will generally be hampered. Older people and persons with dementia cannot rely on facial emotions as before to convey the emotions/intentions of their caregivers nor to express their intentions/emotions in a way that caregivers can usually understand. Consequently, social interaction between these persons will be hindered. Persons might feel isolated, misunderstood and develop depressive symptoms. Note that face masking is only one factor here that adds to social isolation due to measures reducing the risk to be contaminated by COVID-19, such as lock-down procedures, already initiated during the pandemic for disease prevention. The same argument holds true for the following sections.

Impairments of emotion processing and social cognition might not be recognized by relatives and caregivers in older persons due to face masking. Deficits in social communication might further hamper diagnostic workflows and initiation of appropriate treatment. Subjective and mild cognitive impairment—that might increase the risk converting to dementia in the long-term—might not be recognized (Schroeter et al., 2009; Jessen et al., 2014). Accordingly, preventive strategies in older people and cognitively impaired cannot be initiated, such as physical and mental activity, dietary changes and therapy if indicated. Moreover, symptoms of depression and anxiety—as raised by these alterations and potentially unrecognized due to face masking—might accelerate cognitive decline (Santabárbara et al., 2020). We want to underline that these potential relationships as discussed before have to be investigated by new research initiatives.

### Who Will Suffer Most?

One might wonder, whether those deficits in social cognition, i.e., emotion recognition and theory of mind, are a crucial issue for the whole community or just a problem for specific subpopulations. This question has to be answered ambiguously. On the one hand, this issue is relevant for every older subject. As discussed before aging impairs generally social cognition for both, “affective” and “cognitive” theory of mind (Henry et al., 2013) and emotion recognition (Ruffman et al., 2008; Hayes et al., 2020).

On the other hand, specific cohorts need special attention. Older adults with hearing loss—a frequent issue in aging—are prone to problems in social conversation as they cannot anymore rely on their lip-reading skills. This fact is even more important as research has shown an independent association between age-related hearing loss and dementia (Chern and Golub, 2019). Comparable problems might appear in persons suffering from communication problems *per se*, in particular in neurodegenerative primary progressive aphasia (Gorno-Tempini et al., 2011; Bisenius et al., 2016), where its subtypes semantic variant and agrammatic non-fluent variant are subsumed together with behavioral variant frontotemporal dementia under the umbrella term frontotemporal lobar degeneration. Its logopenic variant is rather related to Alzheimer's disease. People with liability to hallucinations/delusions or severe visuo-spatial deficits and prosopagnosia might be unable to recognize persons with face masks correctly, which might be a problem in either Lewy body disease and behavioral variant frontotemporal dementia, or Alzheimer's dementia, posterior cortical atrophy and semantic variant primary progressive aphasia (Neary et al., 1998; Schroeter et al., 2009, 2020; McKeith et al., 2017; Benussi et al., 2021).

Behavioral disease-related impairments might be worsened due to communicative misunderstandings (Schroeter et al., 2011). Further neuropsychiatric diseases need special attention as social cognitive deficits are already specific symptoms of the disease, i.e., in behavioral variant frontotemporal dementia (Pardini et al., 2013; Schroeter et al., 2014, 2018; Bora et al., 2015), or might occur in a broader context of other cognitive symptoms in the most frequent neurodegenerative diseases, i.e., Alzheimer's dementia (Bora et al., 2015), its prestages (Bora and Yener, 2017), and small vessel disease (Kynast et al., 2018). Based on these arguments one might assume that face masking will deteriorate social cognition and disease course in persons with neurodegenerative disease/dementia, which has to be proven in future studies.

Finally, effects of face masking on social behavior might interact with intelligence (Kynast et al., 2020), and gender (Kirkland et al., 2013), where higher intelligence/education, and presumably female sex are expected to strengthen coping strategies.

### Which Strategies Can Minimize Face-Masking Effects on Social Cognition?

Strategies for mental health maintenance shall ensure reliable social cognition, for instance by introducing supportive ways of communication in and with older persons and subjects with dementia (Holmes et al., 2020; Mok et al., 2020). Here, alternative routes of communication might be utilized such as using non-verbal communication, body language, rely on voice characteristics, using more direct verbal communication, taking the social context into account, or even switching to transparent face masks or face shields and telecommunication (Carbon, 2020; Mheidly et al., 2020).

Mok et al. (2020) pronounce the urgent need to establish (cognitive) telemedicine to improve communication with patients and caregivers and related care. We suggest using the term teleconsultation instead as this term includes, beside medicine, also non-medical procedures as performed by psychologists, neuropsychologists, speech, and occupational therapists, as well as social workers. Here, technologies would have to be simplified and infrastructure improved to facilitate widespread videoconferencing. Of note, teleconsultation approaches would guarantee better social communication *via* mask-free facial expressions and enable low-threshold ways of communication, although lack of other communicative channels such as body language and gestures, technical challenges for older persons and sometimes bad quality might be regarded as a drawback. This technology—as well as using transparent masks or any methods that would allow masking without hindering emotions' expression—might even enable continuation of research endeavors, especially as clinical research had to stop in a lot of centers during the COVID-19 pandemic.

Based on the study results of Chaby et al. (2017) investigating gaze strategies in facial emotional recognition with aging, one might also propose adaptive strategies for older persons. If older people rely generally on the lower part of the face to recognize emotions—a maladaptive strategy during face masking—one shall adapt this strategy, refocusing attention on the non-covered upper part of the face.

Of note, persons can apply compensation strategies to cope with limited information from the face only if their other cognitive skills are intact, most importantly executive and language functions. Several conditions affect these executive or control functions early—ranging from (healthy) aging (Schroeter et al., 2003; Maldonado et al., 2020), neurodegenerative diseases such as Alzheimer's dementia (Schroeter et al., 2009, 2012) to small vessel disease (Kynast et al., 2018). People with combined executive deficits and unawareness—particularly in behavioral variant frontotemporal dementia—are unable to even recognize the need for face masking and, consequently, are unable using compensatory strategies (Schroeter et al., 2012, 2014, 2018). Primary progressive aphasia—as discussed above—has a profound impact on language functions (Gorno-Tempini et al., 2011). Hence, executive/language functions and awareness shall be strengthened in people at risk, accompanied by psychoeducation of companions, relatives, and caregivers including compensatory strategies.

## Consequences of Face Masking in The Long Term

In the beginning of the COVID-19 pandemic, governments and people hoped to encounter the disease early. Vaccination strategies have been developed. However, in the meantime, the second or even third pandemic wave have spread. New and prolonged lockdown has been initiated in several countries, where staying at home is recommended except for essential purposes. Simultaneously, face masking had to be prolonged resulting in durations of up to 1 year, e.g. in Germany, where face masking has been practiced continuously in medical and caregiving facilities, public transport, supermarkets and trading.

### Will Face Masking Accelerate Cognitive Decline?

Accordingly, one has to discuss possible long-term effects on older adults and people with dementia in the context of the COVID-19 pandemic. Face masking may impair facial emotion recognition and presumably mindreading (Carbon, 2020), and, herewith, interfere with and disturb socioemotional communication. This might intensify social isolation as discussed before. Indeed, Lara et al. (2019) have shown in a meta-analysis that loneliness is associated with mild cognitive impairment and dementia. As a consequence older people, in particular persons with subjective and mild cognitive impairment as risk states of dementia, might experience cognitive decline (Schroeter et al., 2009; Jessen et al., 2014). Moreover, delayed or even missed diagnosis of cognitive decline due to deficits in socioemotional communication, as discussed before, might hamper recognition and treatment of dementia. Note that impairment in social cognition has been recognized as important for the development of these disorders recently, and included as an additional cognitive domain for the diagnosis of mild and major neurocognitive disorder in the Diagnostic and Statistical Manual of Mental Disorders, 5th Ed. (DSM-5; American Psychiatric Association, 2013). These two diagnostic categories correspond to mild cognitive impairment and dementia. Socioemotional communicative deficits add to the generally reduced utilization of health care services such as day clinics and relief services, visiting general practitioners or taking prescribed therapies as observed in cognitively impaired older persons for instance in Germany during the COVID-19 pandemic (Thyrian et al., 2020). Accordingly, an even accelerated cognitive decline could be expected in older people and cognitively impaired during the COVID-19 pandemic. Moreover, more frequent depression might further accelerate this process. These hypotheses have to be investigated in studies.

### Which Strategies Shall Be Initiated to Improve Social Cognition in the Long Term?

Long-term strategies have to be initiated to ensure sufficient social cognition and prevent cognitive decline. As the impact of face masking with consecutive impaired social cognition is intermingled with the consequences of social isolation, the latter shall be minimized and depression early recognized and treated. Furthermore, proactive strategies have to be designed for early recognition and treatment of deficits in social cognition and beginning cognitive decline. General practitioners might play an important role here, regarding awareness, detection, and home visits.

As a more rapid progression of social cognition impairments and cognitive decline is expected in at risk subjects specific attention has to be given these cohorts. To identify impairments as early as possible, standardized diagnostic tools for social cognition shall be developed and applied in clinical routine with high priority—a neglected cognitive domain so far although included in diagnostic criteria for neurodegenerative diseases already (American Psychiatric Association, 2013; Kynast et al., 2021). Tests shall be adapted for the use in older persons (Kynast and Schroeter, 2018).

Importantly, political measures are required, i.e., ensuring research funding on social cognition and social distress in older adults and (neurodegenerative) diseases during the COVID-19 pandemic, initiating international initiatives to collaborate, raising the general population's and policy makers' awareness about these topics, and guaranteeing state-funded reimbursement of (neuro)psychological consultations.

Finally, early vaccination of older persons and people with dementia is of paramount interest—to prevent COVID-19 in these cohorts and, as an important side-effect, ensure sufficient social communication.

### Will Face Masking Enhance Resilience in the Long Run?

On the other hand, one may take a totally contrary stance. After discussing the drawbacks and shady sides of face masking on social cognition, we will have a look at the other side of the coin—potential assets of face masking for cognition. Face masking might increase the awareness for preventing further spread of the COVID-19 pandemic by sticking to the rules of hygiene. This might be a convincing assumption for well-aging people and so called super-agers, i.e., people aging optimally without essential deterioration in cognitive functions.

Cerami et al. (2020) assessed in the PsyCovid study the influence of psychosocial variables on individual perceived impact of the COVID-19 outbreak in the Italian population. Distress and loneliness were key variables influencing the perceived impact on health. Problem-oriented coping strategies and enhanced empathic abilities increased people's awareness of the severity of the impact of the COVID-19 pandemic on economics. One might argue here that cognitive and emotional resilience might contribute to develop adequate coping strategies.

Another study can also be discussed in this context. Cartaud et al. (2020) investigated whether wearing a face mask to protect from SARS-CoV-2 infection might have an impact on social distancing as another recommended precautionary measure. Surprisingly, interpersonal distance was significantly reduced when characters were wearing a face mask compared to characters displaying neutral, happy or angry facial expressions. Characters with masks were perceived as more trustworthy compared to the other conditions. Interpersonal distance was more diminished in participants infected with SARS-CoV-2 or living in low-risk areas. This study contradicts the initial hypothesis that face masking simply increases the awareness for other prevention measures. It underlines the complex consequences of face masking on emotional and social cognition. However, one might interpret study results by Cartaud et al. (2020) also otherwise, i.e., people are more prone to social distancing when characters are not wearing masks, which seems an expected result of face masking.

## Conclusion

The COVID-19 pandemic will have a high impact on older adults and people with Alzheimer's disease and other dementias. Social cognition enables the understanding of another individual's feelings, intentions, desires and mental states, which is particularly important during the COVID-19 pandemic. To prevent further spread of the disease face masks have been recommended. Although justified for prevention, face masks cover parts of the face and impair recognition of emotions and probably mindreading. As social cognition is already affected by aging and dementia, strategies have to be developed to cope with these profound changes of communication. Face masking even could accelerate cognitive decline in the long run. Further studies are of uppermost importance to address face masks' impact on social cognition in aging and dementia, for instance by longitudinal studies comparing decline before and in the pandemic, and to design compensatory strategies. These issues are also relevant for face masking in general, such as in medical surroundings—beyond the COVID-19 pandemic.

## Author Contributions

MS wrote the first draft and revised the paper with comments and final agreement by all other authors. JK, AV, and SB-C commented. All authors agreed to its final version.

## Conflict of Interest

The authors declare that the research was conducted in the absence of any commercial or financial relationships that could be construed as a potential conflict of interest.

## Publisher's Note

All claims expressed in this article are solely those of the authors and do not necessarily represent those of their affiliated organizations, or those of the publisher, the editors and the reviewers. Any product that may be evaluated in this article, or claim that may be made by its manufacturer, is not guaranteed or endorsed by the publisher.

## References

[B1] American Psychiatric Association (2013). Diagnostic and Statistical Manual of Mental Disorders, 5th Edn. Arlington, VA: American Psychiatric Publishing.

[B2] Baron-Cohen S. Wheelwright S. Hill J. Raste Y. Plumb I. (2001). The “Reading the Mind in the Eyes” test revised version: a study with normal adults, and adults with Asperger syndrome or high-functioning autism. J. Child. Psychol. Psych. Allied Discip. 42, 241–251. 10.1111/1469-7610.00715 11280420

[B3] Benussi A. Premi E. Gazzina S. Brattini C. Bonomi E. Alberici A.. (2021). Progression of behavioral disturbances and neuropsychiatric symptoms in patients with genetic frontotemporal dementia. JAMA Netw. Open 4:e2030194. 33404617 10.1001/jamanetworkopen.2020.30194 PMC7788468

[B4] Bisenius S. Neumann J. Schroeter M. L. (2016). Validating new diagnostic imaging criteria for primary progressive aphasia via anatomical likelihood estimation meta-analyses. Eur. J. Neurol. 23, 704–712. 10.1111/ene.12902 26901360

[B5] Bora E. Walterfang M. Velakoulis D. (2015). Theory of mind in behavioural-variant frontotemporal dementia and Alzheimer's disease: a meta-analysis. J. Neurol. Neurosurg. Psychiatry 86, 714–719. 10.1136/jnnp-2014-309445 25595152

[B6] Bora E. Yener G. G. (2017). Meta-analysis of social cognition in mild cognitive impairment. J. Geriatr. Psychiatry Neurol. 30, 206–213. 10.1177/0891988717710337 28639876

[B7] Carbon C. C. (2020). Wearing face masks strongly confuses counterparts in reading emotions. Front. Psychol. 11:566886. 10.3389/fpsyg.2020.566886 33101135 PMC7545827

[B8] Cartaud A. Quesque F. Coello Y. (2020). Wearing a face mask against Covid-19 results in a reduction of social distancing. PLoS ONE 15:e0243023. 10.1371/journal.pone.0243023 33284812 PMC7721169

[B9] Cerami C. Santi G. C. Galandra C. Dodich A. Cappa S. F. Vecchi T.. (2020). Covid-19 outbreak in Italy: are we ready for the psychosocial and the economic crisis? Baseline findings from the PsyCovid study. Front. Psychiatry 11:556. 10.3389/fpsyt.2020.00556 32587539 PMC7297949

[B10] Chaby L. Hupont I. Avril M. Luherne-du Boullay V. Chetouani M. (2017). Gaze behavior consistency among older and younger adults when looking at emotional faces. Front. Psychol. 8:548. 10.3389/fpsyg.2017.00548 28450841 PMC5390044

[B11] Chen N. Zhou M. Dong X. Qu J. Gong F. Han Y.. (2020). Epidemiological and clinical characteristics of 99 cases of 2019 novel coronavirus pneumonia in Wuhan, China: a descriptive study. Lancet 395, 507–513. 10.1016/S0140-6736(20)30211-7 32007143 PMC7135076

[B12] Chern A. Golub J. S. (2019). Age-related hearing loss and dementia. Alzheimer Dis. Assoc. Disord. 33, 285–290. 10.1097/WAD.0000000000000325 31335455 PMC7749722

[B13] Dodich A. Crespi C. Santi G. C. Cappa S. F. Cerami C. (2021). Evaluation of discriminative detection abilities of social cognition measures for the diagnosis of the behavioral variant of frontotemporal dementia: a systematic review. Neuropsychol. Rev. 31, 251–266. 10.1007/s11065-020-09457-1 33040199

[B14] Ebner N. C. Riediger M. Lindenberger U. (2010). FACES - A database of facial expressions in young, middle-aged, and older women and men: Development and validation. Behav. Res. Methods 42, 351–362. 10.3758/BRM.42.1.351 20160315

[B15] Gorno-Tempini M. L. Hillis A. E. Weintraub S. Kertesz A. Mendez M. Cappa S. F.. (2011). Classification of primary progressive aphasia and its variants. Neurology 76, 1006–1014. 10.1212/WNL.0b013e31821103e6 21325651 PMC3059138

[B16] Grainger S. A. Henry J. D. (2020). Gaze patterns to emotional faces throughout the adult lifespan. Psychol. Aging 35, 981–992. 10.1037/pag0000571 32816505

[B17] Greenhalgh T. Schmid M. B. Czypionka T. Bassler D. Gruer L. (2020). Face masks for the public during the covid-19 crisis. BMJ 369:m1435. 10.1136/bmj.m1435 32273267

[B18] Hayes G. S. McLennan S. N. Henry J. D. Phillips L. H. Terrett G. Rendell P. G.. (2020). Task characteristics influence facial emotion recognition age-effects: a meta-analytic review. Psychol. Aging 35, 295–315. 10.1037/pag0000441 31999152

[B19] Henry J. D. Phillips L. H. Ruffman T. Bailey P. E. (2013). A meta-analytic review of age differences in theory of mind. Psychol. Aging 28, 826–839. 10.1037/a0030677 23276217

[B20] Holmes E. A. O'Connor R. C. Perry V. H. Tracey I. Wessely S. Arseneault L.. (2020). Multidisciplinary research priorities for the COVID-19 pandemic: a call for action for mental health science. Lancet Psychiat. 7, 547–560. 10.1016/S2215-0366(20)30168-1 32304649 PMC7159850

[B21] Jessen F. Amariglio R. E. van Boxtel M. Breteler M. Ceccaldi M. Chételat G.. (2014). A conceptual framework for research on subjective cognitive decline in preclinical Alzheimer's disease. Alzheimers Dement. 10, 844–852. 10.1016/j.jalz.2014.01.001 24798886 PMC4317324

[B22] Kirkland R. A. Peterson E. Baker C. A. Miller S. Pulos S. (2013). Meta-analysis reveals adult female superiority in “Reading the Mind in the Eyes” test. North Am. J. Psychol. 15, 121–146.

[B23] Kynast J. Lampe L. Luck T. Frisch S. Arelin K. Hoffmann K. T.. (2018). White matter hyperintensities associated with small vessel disease impair social cognition beside attention and memory. J. Cereb. Blood Flow Metab. 38, 996–1009. 10.1177/0271678X17719380 28685621 PMC5999004

[B24] Kynast J. Polyakova M. Quinque E. M. Hinz A. Villringer A. Schroeter M. L. (2021). Age- and sex-specific standard scores for the Reading the Mind in the Eyes test. Front. Aging Neurosci. 12:607107. 10.3389/fnagi.2020.607107 33633559 PMC7902000

[B25] Kynast J. Quinque E. M. Polyakova M. Luck T. Riedel-Heller S. G. Baron-Cohen S.. (2020). Mindreading from the eyes declines with aging - evidence from 1,603 subjects. Front. Aging Neurosci. 12:550416. 10.3389/fnagi.2020.550416 33192452 PMC7656776

[B26] Kynast J. Schroeter M. L. (2018). Sex, age, and emotional valence: revealing possible biases in the 'Reading the Mind in the Eyes' task. Front. Psychol. 9:570. 10.3389/fpsyg.2018.00570 29755385 PMC5932406

[B27] Lara E. Martín-María N. De la Torre-Luque A. Koyanagi A. Vancampfort D. Izquierdo A.. (2019). Does loneliness contribute to mild cognitive impairment and dementia? A systematic review and meta-analysis of longitudinal studies. Ageing Res. Rev. 52, 7–16. 10.1016/j.arr.2019.03.002 30914351

[B28] Maldonado T. Orr J. M. Goen J. R. M. Bernard J. A. (2020). Age differences in the subcomponents of executive functioning. J. Gerontol. B Psychol. Sci. Soc. Sci. 75, e31–e55. 10.1093/geronb/gbaa005 31943092

[B29] McKeith I. G. Boeve B. F. Dickson D. W. Halliday G. Taylor J. P. Weintraub D.. (2017). Diagnosis and management of dementia with Lewy bodies: fourth consensus report of the DLB consortium. Neurology 89, 88–100. 10.1212/WNL.0000000000004058 28592453 PMC5496518

[B30] Mheidly N. Fares M. Y. Zalzale H. Fares J. (2020). Effect of face masks on interpersonal communication during the COVID-19 pandemic. Front. Public Health 8:582191. 10.3389/fpubh.2020.582191 33363081 PMC7755855

[B31] Mok V. C. T. Pendlebury S. Wong A. Alladi S. Au L. Bath P. M.. (2020). Tackling challenges in care of Alzheimer's disease and other dementias amid the COVID-19 pandemic, now and in the future. Alzheimers Dement. 16, 1571–1581. 10.1002/alz.12143 32789951 PMC7436526

[B32] Neary D. Snowden J. S. Gustafson L. Passant U. Stuss D. Black S.. (1998). Frontotemporal lobar degeneration: a consensus on clinical diagnostic criteria. Neurology 51, 1546–1554. 10.1212/WNL.51.6.1546 9855500

[B33] Pardini M. Gialloreti L. E. Mascolo M. Benassi F. Abate L. Guida S.. (2013). Isolated theory of mind deficits and risk for frontotemporal dementia: a longitudinal pilot study. J. Neurol. Neurosurg. Psychiatry 84, 818–821. 10.1136/jnnp-2012-303684 23117487

[B34] Peeples L. (2020). What the data say about wearing face masks. Nature 586, 186–189. 10.1038/d41586-020-02801-8 33024333

[B35] Pfefferbaum B. North C. S. (2020). Mental health and the Covid-19 pandemic. New Engl. J. Med. 383:510–512. 10.1056/NEJMp2008017 32283003

[B36] Quesque F. Rossetti Y. (2020). What do theory-of-mind tasks actually measure? Theory and practice. Perspect. Psychol. Sci. 15, 384–396. 10.1177/1745691619896607 32069168

[B37] Ruffman T. Henry J. D. Livingstone V. Phillips L. H. (2008). A meta-analytic review of emotion recognition and aging: implications for neuropsychological models of aging. Neurosci. Biobehav. Rev. 32, 863–881. 10.1016/j.neubiorev.2008.01.001 18276008

[B38] Santabárbara J. Villagrasa B. Gracia-García P. (2020). Does depression increase the risk of dementia? Updated meta-analysis of prospective studies. Actas Esp. Psiquiatr. 48:169–180. 32920782

[B39] Schroeter M. L. (2021). Beyond attention, executive function & memory –resocializing cerebral small vessel disease. Alzheimers Dement. 10.1002/alz.12391 34156144

[B40] Schroeter M. L. Albrecht F. Ballarini T. Leuthold D. Legler A. Hartwig S.. (2020). Capgras delusion in posterior cortical atrophy - a quantitative multimodal imaging single case study. Front. Aging Neurosci. 12:133. 10.3389/fnagi.2020.00133 32547387 PMC7272572

[B41] Schroeter M. L. Laird A. R. Chwiesko C. Deuschl C. Schneider E. Bzdok D.. (2014). Conceptualizing neuropsychiatric diseases with multimodal data-driven meta-analyses - the case of behavioral variant frontotemporal dementia. Cortex 57, 22–37. 10.1016/j.cortex.2014.02.022 24763126 PMC4108513

[B42] Schroeter M. L. Pawelke S. Bisenius S. Kynast J. Schuemberg K. Polyakova M.. (2018). A modified reading the mind in the eyes test predicts behavioral variant frontotemporal dementia better than executive function tests. Front. Aging Neurosci. 10:11. 10.3389/fnagi.2018.00011 29441012 PMC5797534

[B43] Schroeter M. L. Stein T. Maslowski N. Neumann J. (2009). Neural correlates of Alzheimer's disease and mild cognitive impairment: a systematic and quantitative meta-analysis involving 1351 patients. Neuroimage 47, 1196–1206. 10.1016/j.neuroimage.2009.05.037 19463961 PMC2730171

[B44] Schroeter M. L. Vogt B. Frisch S. Becker G. Barthel H. Mueller K.. (2012). Executive deficits are related to the inferior frontal junction in early dementia. Brain 135(Pt 1), 201–215. 10.1093/brain/awr311 22184615 PMC3267982

[B45] Schroeter M. L. Vogt B. Frisch S. Becker G. Seese A. Barthel H.. (2011). Dissociating behavioral disorders in early dementia-an FDG-PET study. Psychiatry Res. 194, 235–244. 10.1016/j.pscychresns.2011.06.009 22044532

[B46] Schroeter M. L. Zysset S. Kruggel F. von Cramon D. Y. (2003). Age dependency of the hemodynamic response as measured by functional near-infrared spectroscopy. Neuroimage 19, 555–564. 10.1016/S1053-8119(03)00155-1 12880787

[B47] Thyrian J. R. Kracht F. Nikelski A. Boekholt M. Schumacher-Schönert F. Rädke A.. (2020). The situation of elderly with cognitive impairment living at home during lockdown in the Corona-pandemic in Germany. BMC Geriatr. 20:540. 10.1186/s12877-020-01957-2 33375944 PMC7770747

